# Bariatric Surgery and Psychological Health: A Randomised Clinical Trial in Patients with Obesity and Type 2 Diabetes

**DOI:** 10.1007/s11695-023-06537-y

**Published:** 2023-03-24

**Authors:** Lynn M. Murton, Lindsay D. Plank, Rick Cutfield, David Kim, Michael W. C. Booth, Rinki Murphy, Anna Serlachius

**Affiliations:** 1 Department of Psychological Medicine, Faculty of Medical and Health Sciences, University of Auckland, 22-30 Park Avenue, Grafton, Auckland, 1023 New Zealand; 2 Department of Surgery, Faculty of Medical and Health Sciences, University of Auckland, 22-30 Park Avenue, Grafton, Auckland, 1023 New Zealand; 3 Department of Endocrinology, North Shore Hospital, Waitemata District Health Board, 124 Shakespeare Road, Takapuna, Auckland, 0620 New Zealand; 4 Department of Surgery, North Shore Hospital, Waitemata District Health Board, 124 Shakespeare Road, Takapuna, Auckland, 0620 New Zealand; 5 Department of Medicine, Faculty of Medical and Health Sciences, University of Auckland, 22-30 Park Avenue, Grafton, Auckland, 1023 New Zealand

**Keywords:** Bariatric surgery, Depression, Anxiety

## Abstract

**Purpose:**

This study investigated the impact of either Roux-en-Y gastric bypass with silastic ring (SR-RYGB) or sleeve gastrectomy (SG) types of bariatric surgery on psychological health and explored the role of pre-existing depressive symptoms on weight loss.

**Materials and Methods:**

A total of 114 participants with obesity and type 2 diabetes were randomized to receive SR-RYGB or SG at a single centre. Data from the Hospital Anxiety and Depression Scale (HADS), RAND 36-item Health Survey and body weight were collected before surgery and annually for 5 years.

**Results:**

Sixteen patients were lost to follow-up at 5 years. Of the 98 patients who completed 5-year psychological follow-up assessments, 13 had mild to severe depressive symptoms (SR-RYGB *n* = *6*, SG *n* = *7*). SR-RYGB and SG resulted in similar psychological health improvement but percent weight loss at 5 years was greater for SR-RYGB by 10.6% (95% CI: 7.2 to 14.0, *P* < 0.0001). Scores for depressive symptoms and most RAND-36 domains improved significantly from baseline to 5 years in both groups. Patients with pre-existing depressive symptoms had similar percent weight loss at 5 years compared to patients without depressive symptoms, irrespective of procedural type.

**Conclusion:**

Patients receiving either SR-RYGB or SG had comparable psychosocial functioning, which was maintained to 5 years post-surgery. Pre-existing depressive symptoms did not affect weight loss achieved at 5 years. These findings confirm previous longitudinal studies demonstrating that bariatric surgery is generally associated with improved psychosocial functioning.

**Graphical Abstract:**

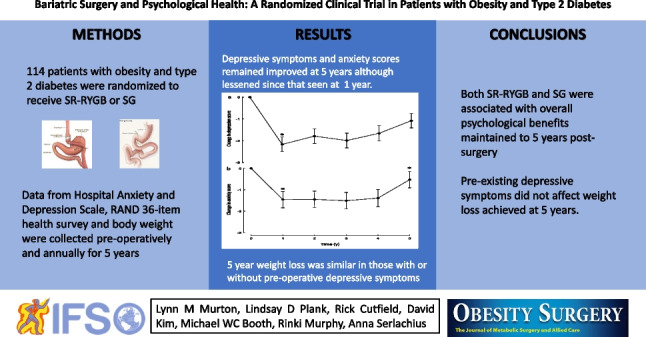

## Introduction

Available research indicates that bariatric surgery promotes psychological improvements in depression, anxiety and quality of life for many patients [1–3]. However, for a minority of patients, psychological health remains unchanged or can even deteriorate over time [4–7]. For example, although several short-term studies have reported that bariatric surgery significantly decreases symptoms of depression and anxiety in the initial post-operative year [8–11], longer-term studies including a recent systematic review[12] have demonstrated that these improvements begin to deteriorate 2 to 3 years post-surgery[5, 9, 10, 13, 14]. Similar questions exist for quality of life outcomes following surgery and whether improvements in health-related quality of life persist over time[15].

Furthermore, there is inconsistent evidence regarding the influence of pre-existing mental health conditions on post-surgical outcomes[16, 17]. While several studies indicate that pre-existing mental health conditions, such as depression and anxiety, contribute to suboptimal weight loss [18–20], others have reported that patients with pre-existing mental health conditions, in particular anxiety and depressive symptoms, have comparable outcomes [21–24]. A recent systematic review categorised risk factors of weight regain after bariatric surgery into five domains: anatomical, genetic, dietary, psychiatric and temporal factors, demonstrating the complex interplay between behavioural, physiological and psychological factors[25].

Despite mixed evidence, several clinical practice protocols typically outline the need for psychological evaluation prior to surgery[26–28]. However, there are currently no specific guidelines pertaining to psychological support prior to or following surgery [29]. Offering psychological support remains a difficult resource issue, given the optimal frequency, timing and duration of sessions that are cost-effective in improving health outcomes is unclear. However, since patients undergoing bariatric surgery have a high prevalence of mental health conditions, some centres offer routine pre- and post-operative psychological support[30].

There is an increasing number of studies exploring the relationship between psychosocial functioning and postoperative psychological and weight outcomes after bariatric surgery[31, 32], however, this has seldom been investigated in a prospective randomised blinded study comparing two different procedure types, or investigated in a New Zealand population. While the demand for bariatric surgery continues to rise, these procedures remain a scarce resource, with relatively low numbers of public-funded and private-funded procedures occurring relative to the large numbers of patients who could benefit from this[33, 34]. Therefore, it is important to determine both the physiological and psychological benefits of the different types of bariatric surgery in patients with obesity and type 2 diabetes in order to prioritise these procedures to patients who will benefit the most [2, 35].

This study aimed to investigate the long-term psychological impact of Roux-en-Y gastric bypass with silastic ring (SR-RYGB) and sleeve gastrectomy (SG) in a multi-ethnic cohort of patients with obesity and type 2 diabetes in New Zealand, within a randomised, double-blind trial design [36] in which routine pre- or post-operative psychological support was not provided. Firstly, the study investigated the psychological impact of bariatric surgery over time and whether this differed by type of procedure. Secondly, this study explored the relationship between pre-surgical depressive symptoms and weight loss up to 5 years post-surgery.

## Material and Methods

### Study Design and Population

The trial rationale, protocol[36] and one-year results have been previously reported[8]. In brief, the trial was a randomised, two-arm, double-blind, single-centre study in Auckland, New Zealand, involving 114 patients with obesity and type 2 diabetes. The primary outcome was diabetes remission at 5 years[37]. The current secondary analysis aimed to investigate the impact of SR-RYGB and SG on depressive symptoms, anxiety symptoms and quality of life, as well as the role of pre-surgical psychological health on bariatric surgery outcomes, analysed up to 5 years post-surgery. Ethics approval was obtained from the New Zealand Regional Ethics Committee (NTY/11/07/082) and written informed consent was obtained from all of the participants. The trial was registered at ANZCTR (ACTRN12611000751976) and clinicaltrials.gov (NCT01486680). Recruitment and randomization were performed as previously described [36]

Eligible participants met the following criteria: (1) aged between 20 to 55 years; (2) BMI of 35 to 65 kg/m^2^ for at least 5 years; (3) type 2 diabetes diagnosis for a minimum of 6 months; (4) suitable to receive either SR-RYGB or SG; and (5) available for the follow-up period. Criteria for exclusion were: (1) postprandial C peptide < 350 pmol/L; (2) pregnant; (3) current smoker; (4) diagnosis of type 1 diabetes or secondary diabetes; (5) chronic pancreatitis; (6) using oral steroid therapy; and (7) not suitable for general anaesthesia.

### Procedures

Participants were admitted to hospital the night before surgery and were randomised to receive either SR-RYGB or SG on the day of surgery. Both procedures were performed laparoscopically as previously described[36]. Participants remained in hospital for approximately 1–2 days post-surgery and were discharged when the medical team deemed suitable. Participants were provided information about postoperative dietary requirements, including the use of Optifast, multivitamins and antacid medication. Clinical assessments, body weight measurements and questionnaires were completed at baseline (pre-surgery), and at 1, 2, 3, 4, and 5 year follow up appointments. Sociodemographic and clinical information were collected at baseline.

### Psychological Health Outcomes

Depressive and anxiety symptoms were assessed using the Hospital Anxiety and Depression Scale (HADS) [38]. The questionnaire consists of 7 items assessing depressive symptoms and 7 assessing anxiety symptoms on a 4-point scale ranging from 0 to 3. Both measures therefore have a maximum score of 21 with higher scores representing higher levels of anxiety and/or depressive symptoms. The HADS is reported to be a reliable instrument for identifying depressive and anxiety symptoms in a range of population groups[39]. In patients undergoing obesity surgery it has shown good responsiveness to change[6]. Scores between 0 and 7 indicated no depressive symptoms and scores of 8 and above mild or severe symptoms. Health-related quality of life was assessed using the RAND 36-item Health Survey [40] which evaluates quality of life in 8 domains comprising physical functioning, role limitations caused by physical health problems, emotional well-being, role limitations caused by emotional problems, social functioning, energy/fatigue, bodily pain, and general health. Scores range from 0 to 100, with higher scores reflecting better quality of life. This instrument is identical to the medical outcomes study 36-item short form (SF-36)[41], and has been validated across a range of chronic conditions[42] and in obese patients[43].

### Statistical Analysis

Data are presented as means and standard deviations unless stated otherwise. Between- and within-group comparisons were carried out using Student’s t test, unpaired and paired respectively. Longitudinal analyses were conducted on all available data using linear mixed-effects models for repeated measures with changes from baseline as dependent variable, surgery group, time and time-by-group interaction as fixed effects and participants as a random effect. Bivariate associations were assessed using Spearman’s rank correlation. Two-sided *P* values < 0.05 were considered to indicate statistical significance. All analyses were carried out using SAS v9.4 (SAS Institute, Cary, NC).

## Results

Of the 114 participants completing baseline assessments for this trial, 98 completed psychological assessments at 5 years, 49 receiving SR-RYGB and 49 SG. There were two deaths among the non-completers and, of the remainder, four were not contactable, nine declined and one did not complete the psychological assessment. Baseline characteristics are presented in Table 1 where it can be seen that the two groups were well-balanced for these characteristics. Regarding HADS scores at baseline, 6 participants (12%) in the SR-RYGB group scored over the cut-off for mild to severe depressive symptoms, and 11 participants (22%) scored over the cut-off for mild to severe anxiety symptoms. In comparison, 7 participants (14%) in the SG group scored over the cut-off for mild to severe depressive symptoms, and 14 (29%) scored over the cut-off for mild to severe anxiety symptoms.

**Table 1:** Baseline characteristics of the study participants

Variable	Gastric bypass (n = 49)	Sleeve gastrectomy (n = 49)
Age (years)	47.9 ± 5.8	46.9 ± 6.1
Sex (male), n (%)	21 (44)	26 (53)
Ethnicity, n (%)		
Māori	8 (16)	8 (16)
Pasifika	6 (12)	2 (4)
NZ European	30 (61)	34 (69)
Other	5 (10)	5 (10)
Weight (kg)	123.0 ± 22.3	124.4 ± 23.5
BMI (kg/m^2^)	41.9 ± 6.0	42.3 ± 6.1
T2D Duration (years)	7.0 ± 5.6	6.8 ± 5.1
HADS		
Depressive Symptoms Score	3.3 ± 2.8	3.6 ± 3.3
Anxiety Symptoms Score	4.7 ± 3.4	6.0 ± 4.8
HADS Scoring Criteria		
Mild to Severe Depressive Symptoms, n (%)	6 (12)	7 (14)
Mild to Severe Anxiety Symptoms, n (%)	11 (22)	14 (29)
RAND-36		
Physical Functioning	67.8 ± 24.5	74.1 ± 21.1
Role Limitations due to Physical Health	71.4 ± 37.5	73.4 ± 37.7
Role Limitations due to Emotional Problems	87.1 ± 27.9	83.0 ± 32.5
Energy/Fatigue	59.6 ± 21.2	58.1 ± 19.4
Emotional Well Being	84.1 ± 12.7	78.2 ± 18.6
Social Functioning	82.4 ± 23.2	82.8 ± 21.4
Bodily Pain	72.4 ± 23.8	75.5 ± 22.0
General Health	53.9 ± 24.2	55.1 ± 23.7

Data presented as mean ± SD

### Impact of SR-RYGB and SG on Psychological Health

**Fig. 1:**
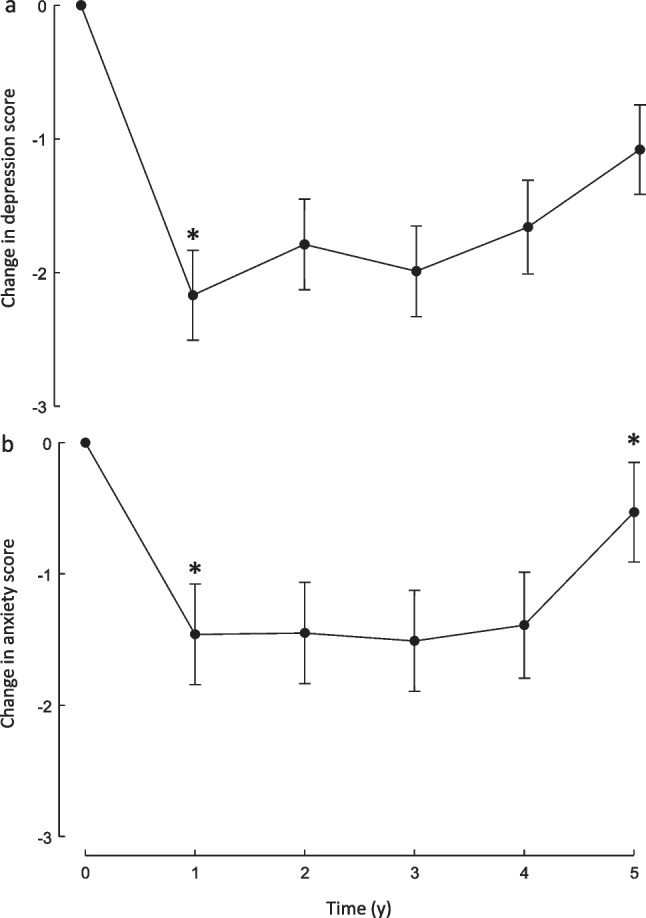
Depression and anxiety scores over 5 years of follow-up in participants undergoing silastic ring Roux-en-Y gastric bypass or sleeve gastrectomy. Changes from pre-surgery (time 0) for depressive symptoms (a) and anxiety symptoms (b) at annual post-surgery follow-up assessments. Data are means ± SEM. *P < 0.05 vs preceding measurement

**Fig. 2:**
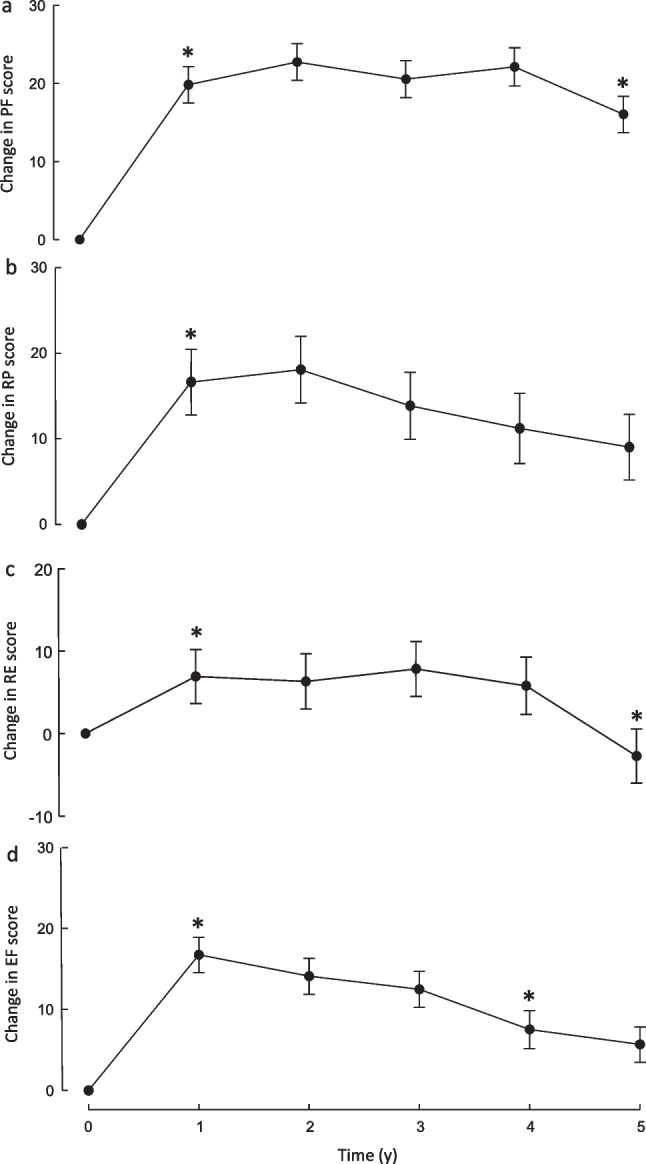
RAND-36 domain scores over 5 years of follow-up in participants undergoing silastic ring Roux-en-Y gastric bypass or sleeve gastrectomy. Changes from pre-surgery (time 0) for physical functioning (PF, a), role limitations caused by physical health problems (RP, b), role limitations caused by emotional problems (RE, c), energy/fatigue (EF, d), emotional well-being (EW, e), social functioning (SF, f), bodily pain (BP, g), and general health (GH, h) at annual post-surgery follow-up assessments. Data are means ± SEM. *P < 0.05 vs preceding measurement

For the changes from baseline over the 5-year period of follow-ups there were no significant surgery group-by-time interaction effects for any of the psychological health variables (all P > 0.2) and, therefore, the impact of bariatric surgery on psychological health was analyzed for the sample as a whole (Figs. 1 and 2). Depressive symptoms significantly improved from baseline to 1 year (*P* < 0.0001), following which there were no significant changes between 1 to 2, 2 to 3, 3 to 4, and 4 to 5 years. However, between 3 and 5 years, depressive symptoms increased significantly (P = 0.015) but remained lower than baseline at 5 years (P = 0.005). A similar pattern was seen for anxiety symptoms except that between 4 and 5 years anxiety symptoms increased significantly (P = 0.035) to reach a level not significantly different from baseline (P = 0.25). Significant increases over the first year were seen for RAND-36 scores for physical function (P < 0.0001), role limitations due to physical health (P < 0.0001), role limitations due to emotional problems (P = 0.027), energy/fatigue (P < 0.0001), bodily pain (P < 0.0001), and general health (P < 0.0001). At 5 years, physical function (P < 0.0001), role limitations due to physical health (P = 0.019), energy/fatigue (P = 0.014), bodily pain (P = 0.003), and general health (P < 0.0001) remained higher than baseline. No significant changes in emotional wellbeing were seen over the 5-year period. Social functioning declined between years 4 and 5 (P = 0.013) but at 5 years did not differ significantly from baseline (P = 0.23).

### Body Weight Changes

**Fig. 3:**
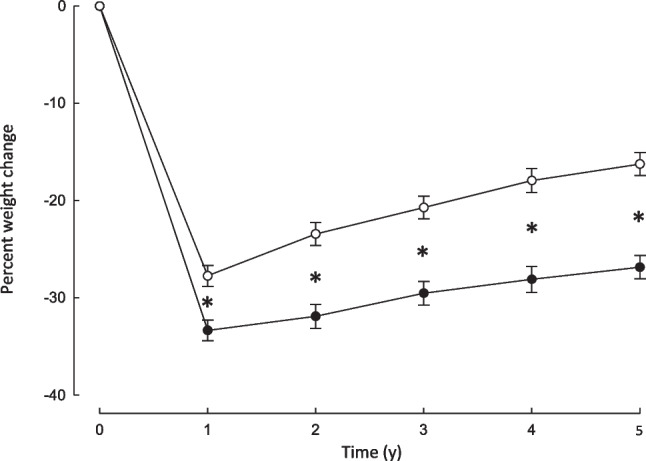
Percent body weight loss over 5 years of follow-up in participants undergoing silastic ring Roux-en-Y gastric bypass or sleeve gastrectomy. Percent losses from pre-surgery (time 0) in body weight at annual post-surgery follow-up assessments in patients who underwent laparoscopic gastric bypass (closed symbols) or laparoscopic sleeve gastrectomy (open symbols). Data are means ± SEM. *P* < 0.0001 for comparison of the time profiles (time x surgery interaction) using a repeated-measures mixed model. *P < 0.001 for between-group comparisons at each time point

Percent losses in body weight from baseline are shown in Fig. 3 for the two surgical groups at each follow-up time point. Percent weight loss at 1 year was less for SG than for SR-RYGB patients (27.8 ± 7.5 vs 33.4 ± 7.4, P = 0.0004). Over the 1 to 5-year period, percent weight gain for SG patients was greater than for SR-RYGB patients (16.8 ± 13.4 compared to 9.9 ± 10.5; P = 0.006).

### Pre-surgical Depressive Symptoms and Post-surgical Weight Change

**Fig. 4:**
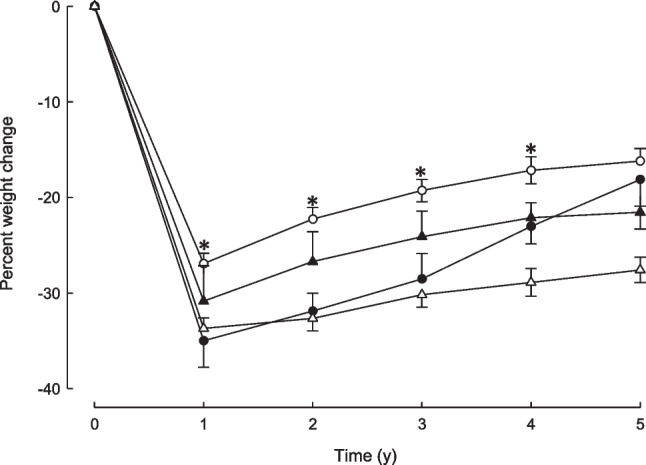
Percent body weight loss from pre-surgery (time 0) over 5 years of follow-up in participants with mild to severe depressive symptoms (closed symbols) or no depressive symptoms (open symbols) at baseline undergoing silastic ring Roux-en-Y gastric bypass (triangles) or sleeve gastrectomy (circles). Data are means ± SEM. For participants who underwent laparoscopic gastric bypass: *P* = 0.348 for time x group interaction, *P* < 0.0001 for time effect, *P* = 0.061 for group effect. For participants who underwent laparoscopic sleeve gastrectomy: *P* = 0.0009 for time x group interaction, *P < 0.030 for between-group comparisons at each time point

Those with mild to severe depressive symptoms at baseline, compared to those without depressive symptoms, trended to lower percent weight loss after SR-RYGB at all time points although this was not statistically significantly different (Fig. 4). For SG patients, those with mild to severe depressive symptoms at baseline, achieved greater percent weight loss initially at 1 year (35.0 ± 6.8 vs 26.9 ± 7.0, p = 0.011), but thereafter, their weight regain was much greater as shown in Fig. 4. By 5 years after SG there was no difference in percent weight loss achieved between those with and without mild-severe depressive symptoms at baseline (18.1 ± 7.4 vs 16.2 ± 8.3, respectively, P = 0.57). Baseline depressive symptoms were positively correlated with weight regain between 2 and 5 years for SG patients (*r* = 0.44, *P* = 0.002) but not for SR-RYGB patients (*r* = –0.11,* P* = 0.44). Weight regain over this period for those with and without mild-severe depressive symptoms at baseline was 5.4 ± 5.9 vs 6.5 ± 6.2 kg for SR-RYGB (P = 0.68) and 17.8 ± 12.2 vs 8.1 ± 8.2 kg for SG patients (P = 0.030).

## Discussion

In patients with obesity and type 2 diabetes, both SR-RYGB and SG were associated with significant improvements to depressive symptoms and quality of life scores from baseline to 5 years. The majority of this overall improvement occurred in the initial post-operative year when the greatest weight loss also occurred. Although improvements to depressive symptoms and quality of life plateaued after the first year, these remained as significant improvements from baseline at 5 years. In contrast, despite a significant improvement in anxiety symptoms from baseline to 1 year, this returned to baseline levels at 5 years. Pre-existing depressive symptoms tended to be associated with lower percent weight loss after SR-RYGB, but this was not significantly different at 5 years. Those with pre-existing depressive symptoms had greater weight loss at 1 year after SG, but then greater weight regain between 2 and 5 years, leading to similar weight loss at 5 years.

Previous studies have indicated that both SR-RYGB and SG procedures produce similar improvements to depressive symptoms, anxiety symptoms[8, 11, 13], and health-related quality of life[44–46]. In line with the current study, research indicates that these improvements are most prominent in the first 1 to 2 years following surgery, but often deteriorate over time [6, 10, 13, 47]. Although the mechanisms influencing this pattern are unclear, literature suggests that changes to mental health may be linked to changes in weight [24, 48, 49]. In the present study, the majority of weight loss occurred in the first post-operative year, mirrored by a significant improvement in psychological health. However, following this initial trend, changes to psychological health and weight no longer appeared to share similar trajectories. These findings are consistent with previous research which suggest that changes to mental health and quality of life do not directly correspond to the degree of weight loss[9, 11, 13, 50].

In line with previous research, both surgeries facilitated an improvement to the physical functioning, role limitations caused by physical health problems, energy/fatigue, bodily pain, and general health domains from baseline to 5 years, but there was no evidence of improved emotional wellbeing[8, 10, 51–53]. Given bariatric surgery produces significant weight loss, it is not surprising that there are greater benefits to the physical quality of life domains, however, this does highlight the need to address what factors are preventing similar improvements to emotional wellbeing[53–55]. Previous bariatric research has suggested that post-surgical mood disorders contribute to suboptimal improvements to emotional wellbeing/mental quality of life domains[9]. This may also have been the case in the current sample, as after the initial decline in depressive symptoms and anxiety symptoms, there was an upwards trend from 3–5 years, with anxiety reverting to pre-surgery levels.

Anxiety symptoms may be less likely to improve in comparison to depressive symptoms as a result of bariatric surgery, because although often overlapping disorders, they are inherently different with distinct psychological and physical symptoms. Depressive symptoms (in particular anhedonia which the HADS taps into) may be more likely to improve after bariatric surgery due to the well-established bidirectional association between depression and obesity[56]. Furthermore, it is likely that losing weight after bariatric surgery may increase behavioural activation, that is increasing the behaviours which lead to increased positive emotions and pleasure (e.g. increased social activities). Anxiety on the other hand, in particular symptoms of generalised anxiety disorder which the HADS measures, may not be directly implicated or may be negatively implicated by bariatric surgery.

Literature exploring the impact of depressive symptoms on bariatric surgery outcomes is so far inconclusive[16, 17]. The current study found those with known depressive symptoms who received SG had greater weight loss initially which was followed by greater weight regain between 2 and 5 years, resulting in similar overall weight loss outcomes at the 5-year follow-up compared to those without depressive symptoms. This time course data following SG is in contrast to several other studies which have reported similar weight loss outcomes among those with and without depressive symptoms[21–23]. Several other longitudinal studies have found that patients who report higher levels of depressive symptoms at baseline are at a greater risk of long-term weight regain following surgery [9, 57, 58]. One possibility that pre-existing depressive symptoms is associated with weight recidivism may be explained by the pervasive nature of depression. For instance, in the initial post-operative year depressive symptoms subside in parallel with weight loss[5, 13, 48]. However, as weight loss begins to subside, depressive symptoms return, contributing to adverse behaviours associated with weight regain[59]. In the current study, those with mild to severe depressive symptoms at baseline who received SR-RYGB had a trend to lower percent weight loss at 5 years, compared to those without depressive symptoms at baseline. This was due to slightly lower percent weight loss achieved at all earlier time points, without any difference in weight regain between 2 and 5 years, such that weight loss was similar at 5 years. Weight regain may have been prevented by the SR-modification of the RYGB, as the placement of a silastic ring around the gastric pouch prevents stomal dilation which is frequently associated with weight regain after RYGB and SG[60].

Higher prevalence of pre-existing mental health issues in bariatric surgery candidates, may reflect this being one of the reasons driving people to seek bariatric surgery[61]. Previous studies have explored patient expectations prior to bariatric surgery and have found that patients often hold unrealistic expectations regarding guaranteed and enduring weight loss following surgery [62, 63]. One possibility for the plateau in quality of life and psychological outcomes after the first year and subsequent weight regain could in part be due to patient misconceptions that surgery will ‘cure’ their obesity and associated comorbidities, including possible mental health comorbidities. These expectations can have a detrimental impact on psychological health when weight regain does occur[64], further highlighting the importance of pre and post-operative education and psychosocial support.

One of the most significant contributions of this study is the clinical implications these findings may have on the provision of psychological support throughout bariatric surgery treatment. Most bariatric services require a psychological evaluation prior to weight loss surgery [26–28]. However, there are currently no existing guidelines that specify the provision of psychological care prior to, or following, bariatric surgery[29]. Psychological support was not offered as a routine part of bariatric care in this study. Given access to weight loss surgery is already constrained by available resources and funding[33], it is important to determine the most effective way to implement psychological support. The findings from the present study suggest that patients presenting with self-reported mild to severe depressive symptoms benefit from similar weight loss long term after surgery to those without these symptoms. However, it is important to note that previous studies have shown that those patients who died by suicide or had non-fatal self-harm events after bariatric surgery, had similar or lower body weight during follow up than patients who did not[65]. Depressive symptoms have been linked with suicide, and while absolute risk of suicide is low, the relative risk of suicide has been demonstrated to be higher after surgery (2.6 vs 0.9 per 10,000 person years of follow up)[65]. While reassuring, average improvements in health-related quality of life or depressive symptoms may mask deteriorating levels in a small subset who may be at risk of suicide or self-harm events. Further studies evaluating whether psychological support, particularly in the later post-operative years, improves long-term weight maintenance and psychological health are required.

Although this study has several strengths, there were also some limitations. Firstly, this study did not include those with severe clinical depression so our conclusions are not generalisable to this group. In addition to this, indicators of pre-operative depressive symptoms were dependent on self-reported measures and previous research indicates that bariatric patients may underreport symptoms to avoid exclusion from surgery[66, 67]. Thirdly, we did not have a non-surgical control group so we cannot exclude the possibility that participants’ depressive symptoms improved over time rather than due to surgery. Lastly, although the single-centre design facilitates minimal variability, it also reduces generalisability, which may be viewed as a limitation. Despite these limitations, a key strength of this study is that it is one of the largest double-blind randomised trials comparing the efficacy of SR-RYGB and SG in patients with obesity and type 2 diabetes.

## Conclusions

The current study demonstrated that SR-RYGB and SG were both associated with significant benefits in depressive symptoms and the physical quality of life domains, maintained up to 5 years. Yet, despite initial improvement to anxiety symptoms, this was not sustained long-term. While those with modest pre-surgical depressive symptoms achieved differential patterns of weight loss by surgical type, by 5 years the percent weight loss after either SR-RYGB or SG was not significantly different between those who did and did not have pre-surgical depressive symptoms.
